# Supplementary material for: COVIVA: Effect of transcutaneous auricular vagal nerve stimulation on fatigue-syndrome in patients with Long Covid – A placebo-controlled pilot study protocol
Source: PLoS One. 2025 May 9;20(5):e0315606. doi: 10.1371/journal.pone.0315606 (PMC12063903; doi:10.1371/journal.pone.0315606)
Supplement: Attachment — Submitted filename: Comments to the Reviewer.docx [file pone.0315606.s004.docx]

We thank the reviewers and the editor for the interesting and thoughtful comments and especially the mentioned internet resource OSF.

Editor:

**When completing the data availability statement of the submission form, you indicated that you will make your data available on acceptance. We strongly recommend all authors decide on a data sharing plan before acceptance, as the process can be lengthy and hold up publication timelines. Please note that, though access restrictions are acceptable now, your entire data will need to be made freely accessible if your manuscript is accepted for publication. This policy applies to all data except where public deposition would breach compliance with the protocol approved by your research ethics board. If you are unable to adhere to our open data policy, please kindly revise your statement to explain your reasoning and we will seek the editor's input on an exemption. Please be assured that, once you have provided your new statement, the assessment of your exemption will not hold up the peer review process.**

This is only a study protocol, so there is no additional data which could be published at this point. Once the study is completed and analyzed, the results will be made available within the publication of the results.

**Reviewer #1: Thank you for the opportunity to review this paper. This is an interesting manuscript presenting a protocol of placebo-controlled pilot study on effect of transcutaneous auricular vagal nerve stimulation on the fatigue syndrome. The primary objective of this study is to investigate the effect of taVNS on patients with long Covid fatigue syndrome.
My review mainly concerns only the statistical aspects of the study. Some questions reported below were raised and in my view, it is not acceptable in this form for the publication in this journal.

1. The sample size justification reported is incomplete in relation to primary outcame. Is not indicate the primary end-point variable on which the estimation is evaluated; the common SD estimated is not reported;**

Statistically, the difference between the two groups is checked in continuous data (main target parameters and ranked judgment scales) using exact rank variance analysis according to Kruskal and Wallis (R package “coin”) to produce a 2-sided p-value. The threshold for the p-value is an alpha of 0.05, i.e. if the test value falls below the threshold, the null hypothesis can be rejected and a significant general difference between the three groups (placebo, low-threshold, higher-threshold group) can be assumed. In addition, the two treatment groups are each compared descriptively with placebo (and with each other). Secondary target parameters are the BDI, the SF-36 and acceptance; these are upgraded in analogy to the main target parameter. Since exactly one main target parameter (MFI20 change) is evaluated for confirmatory purposes, no adjustment is necessary Type 1 risk is necessary. Based on the expectation of superiority of the two target groups (low and higher threshold stimulation) of at least 5 or 10 MFI units compared to placebo (shift) and a type 1 error of alpha = 0.05, a total of 3 x 12 cases = 36 cases in total needed to achieve a study power of 80%. The numbers mentioned are minimum numbers and should be secured by a safety surcharge (depending on the drop-out rate) of around 10% - 20%, so that (if the surcharge is necessary) this results in a total of 3 x 15 cases = 45 cases in total. The calculation was carried out using the R package “MultNonParam” (Kolassa & Seifu, 2013). The remaining parameters (BDI, SF-36, acceptance judgment) are evaluated purely descriptively at the threshold of p = 0.05 (same statistical test as the main target parameter ). The calculated p-values are always two-tailed. Means, medians, quantiles and the respective sample numbers of the available data are given descriptively. For the between-group contrast, the mean difference and its 95% confidence intervals are given as the effect measure. In addition, a nonparametric estimate of the group effect (shift) using the Hodges-Lehman estimator shift (Lehmann, 1998) is given

**2. In the statistical analysis paragraph should be more detailed the model applied for the analysis with relative post-hoc analysis;**

Since there is a single primary target parameter the confirmatory analysis will not be adjusted for multiple testing. All other parameters will be tested descriptively only (generating hypotheses only).

**3. There are some error of typing. Please, verify the manuscript for English and typos.**

We had the manuscript proofread.

**Reviewer #2: Summary
This paper presents the COVIVA study protocol, which aims to evaluate the feasibility of transcutaneous auricular vagal nerve stimulation (taVNS) as a treatment for patients with long covid syndrome and its potential to alleviate fatigue. The description of the planned study is informative but requires additional details and justification to ensure reproducibility. For example, the statistical test employed for the sample size calculation and the theoretical effect size underlying this calculation need to be clearly outlined. The findings of this study have the potential to guide the development of taVNS as a treatment for patients with Long-COVID syndrome. Therefore, this protocol is worthy of publication, provided that the methods and materials are thoroughly revised and my major comments are adequately addressed.
Major comments
1. The theoretical foundation of developing taVNS as a treatment for covid- associated fatigue in this study is its anti-inflammatory effect. While the anti-inflammatory effect of taVNS is well-established, it remains unclear whether taVNS alleviates fatigue through the anti-inflammatory reflex. To address this, researchers must examine the relationship between fatigue and inflammatory cytokines, which is not the case in reference [15], [35] and [36]. Note that reference [10] states that elevated IL-10 and IL-6 are important at the early stage but do not affect persistent fatigue once the fatigue syndrome is established. Another study has attempted to investigate whether taVNS improves fatigue through modulating inflammatory markers but found no correlation between fatigue and proinflammatory cytokines (please take a look at their results and discussion https://www.neuromodulationjournal.org/article/S1094-7159(22)01263-6/fulltext ) They stated “it has been established that VNS including nVNS modulates EEG microstates and brain activity”, suggesting VNS improving fatigue might be a central mechanism. This is supported by a behavioral and physiological study of the effect of taVNS on fatigue (https://www.brainstimjrnl.com/article/S1935-861X(24)00060-3/fulltext), which found that the taVNS modulates the arousal pathway and rescue fatigue at the later stage of memory tasks. This is a more direct evidence to support the use of taVNS in patients with long-covid syndrome in comparison to studies about its anti-inflammatory effects.**

We corrected this accordingly and added respective notes and explanations.

**In the current manuscript, the link between fatigue and inflammation after COVID-19 infection and the anti-inflammation effects of taVNS is missing. I suggest that the author introduces taVNS as a potential intervention after line 84, The introduction of taVNS should include its history (current line 98 - 110) and current knowledge about its positive effects on fatigue such as the results from the aforementioned studies. The authors should then elaborate on the potential mechanism of fatigue and the mechanism of taVNS. Please verify if the inflammation persist months after the infection of COVID-19 potentially leading to fatigue because intuitively, the inflammation might disappear when the patient recovered from COVID-19. Again, based on the current literature, the effect of taVNS on neuroepinepherine is more likely to be the mechanism for its positive effect on fatigue, if there is any.**

We corrected this accordingly.

**The authors state, “Due to the current pandemic, further research on the causes of fatigue has not yet been conducted, and the underlying cause of fatigue remains inconclusive.” The current flow of the introduction implies that this protocol will test whether taVNS can mitigate fatigue through the regulation of pro-inflammatory cytokines, which is not the case. Please restructure the introduction to focus more on the effects of taVNS on fatigue and its feasibility. For instance, what is the significance of establishing the feasibility of taVNS in patients with long-COVID syndrome? Does taVNS need to be administered for a specific duration to treat fatigue?**

The reason to investigate feasibility is to find out whether patients with severe fatigue are even able to use taVNS for 4 hours per day. Our experience with the first patients is that having something to do for 4 hours can be a burden for these patients.

**Please avoid statements such as “In summary, vagus nerve stimulation appears to have the potential to reduce the occurrence of fatigue and depression in long Covid syndrome through the reduction of cytokines, or treat these symptoms.”**

We corrected this.

**2. More details about the methods need to be provided. For example, Line 130. Fatigue persisting for >= 3 months. Is it a subjective report of fatigue? Please introduce the fatigue scale in the introduction. In the exclusion criteria, how to define acute Covid infection. 14 days within the infection? What is Vagus nerve stimulation (line 139) in the exclusion criteria? Do you mean having done a surgical procedure for implanting the cervical VNS device? Please make the inclusion/exclusion criteria as objective as possible. I suggest consolidating Questionnaires: and endpoints (line 167) into one section: primary outcomes.**

We added the scale and the respective explanatations as suggested. Our ethics committee required us to define the acceptance rate as the primary outcome and the questionnaires as the secondary outcome.

**One of the outcomes is average stimulation duration. Is the total stimulation duration controlled? Will the patient receive instructions about the stimulation duration? Second outcome is MF120, as this is the metric that the author will use to calculate sample sizes. Please clearly state that analysis of other questionnaires is considered exploratory analysis, and the exploratory result should not be used to draw conclusion once the study is completed.**

The patients will be instructed, we added a respective paragraph. MFI20 is a secondary outcome – the primary outcome is acceptance.

**The sample size calculation needs to be more specific. To calculate the sample size, one will need to define the test that will be used, the power that needs to be reached, and the theoretical effect size. Line 202 “The group size is based on the aforementioned studies on taVNS in other forms of fatigue 202 (29,36,37), our aforementioned FatiVa study (38),” Simply using the number of patients of previous studies is not a scientific way for defining sample size because previous studies can be under-powered. “The threshold for the p-value is an alpha of 0.05,” which can be called the significance level.**

**Line 213 “Based on the expectation of a superiority
213 of the two target groups (low-threshold and high-threshold stimulation) of at least 5 and 10 MFI
214 units, respectively, compared to placebo (shift), and a type I error of alpha = 0.05, a total of 3
215 x 12 cases = 36 cases are required to achieve a study power of 80% in the most important
216 secondary endpoint, the MFI20 questionnaire. The” What is the statistic test that will be used? Is there any evidence for the expectation of 5-10 MFI units?**

Please refer to the yellow section above, there specifics and details of the sample size estimation.

**Could you explain what successful acceptance is and how it will be used? “Successful acceptance and compliance are achieved when the acceptance criteria, as defined
228 in the primary endpoint, are met by at least 80% of patients.” What is the acceptance criteria? Do you mean if more than 80% of patients use taVNS for more than 4 hours, then taVNS is feasible?**

Yes, we corrected that.

**Line 264, it seems that this study includes a titration of intensity. Please state how the investigator will reduce the intensity from the initial setting, for example, with a decrement of 5 units when the patient reports feeling the stimulation. How do you ensure that the Verum -1 group uses the predefined stimulation intensity?**

We corrected that – the patients will repeat the procedure to actively downregulate the intensity below threshold.

**In study plan, the study started last year. Have any patients been recruited? Is there any preliminary data that can be used to estimate the effect size?**

We have not yet performed any analysis as the data is still “sealed” in our Recap-System. It will be analyzed once the study is completed.

**3. In conflict of interest, as “MG is founder and advisor of Neuroloop GmbH,” The authors need to state whether this company will manufacture products to reduce fatigue in patients with long-covid syndrome.**

This statedment was added.

**4. In the discussion section, fatigue is stated as one of the side effects of taVNS in a meta-analysis published in a scientific report. Please discuss whether this side effect will confound the analysis.**

The only side effect of taVNS close to fatigue is dizziness, which means vertigo in this context. Fatigue is no known side effect of taVNS.

**5. Please verify if cymba conchae, rather than tragus, is most innervated by the auricular branch of the vagus nerve. (https://www.ncbi.nlm.nih.gov/pmc/articles/PMC7083568/). In reference [22], it seems that they also indicates that tragus is partly innervated by the auricular branch of the vagus nerve. The authors might need additional evidence to support stimulation of aVN through tragus, for example, tragus is easier to access.**

We corrected this, it was our fault – our system stimulates the cymba conchae which is innervated by the auricular branch of the vagal nerve. We added the respective literature.

Minor comments
**1. Abstract, first sentence, I understand this data comes from study. But to provide a background for this protocol, I suggest that the authors state for example, estimated number of patients who is currently having long covid syndrome.**

We added a respective link to the WHO, stating that 775 mio. Patients suffered from Covid-19. If only 10% of them get long covid, it is more than 77mio patients.

**2. Page 3, line 70, “In addition to fatigue (58%), these patients experience headaches
(44%), anosmia, anxiety (13%), and depression (12%). Please specify the denominator used to calculate these percentages.**

We corrected this an just referred to one study with 143 patients.

**3. Key message, page 3, is the key message section required to be published in PLOS ONE? Key messages in the manuscript seem to be a shorter abstract. The manuscript without the key messages is complete.**

We deleted them.

**4. Please rephrase sentences 81 – 83; for example, ‘fatigue can continue to affect these patients months after recovery from the infection.’**

We rephrased the sentence.

**5. The use of abbreviations needs to be consistent. For example, line 115 is transcutaneous VNS taVNS?**

There are other transcutaneous systems stimulating the vagle nerve directly through the skin. We change “transcutaneous” to “non-invasive” VNS to make this more clear.

**6. What does Verum stand for?**

“Verum” is the latin word for “true”, which means in this context “true stimulation group”.

**7. Change references into English. Are the journal name and publication date in Germany?**

Yes, sorry, we corrected that.

**8. Optional: provide instruction to the three groups of patients as supplementary material.**

The patients receive the official manual of the stimulator and a verbal instruction of the system. The stimulator is very easy to use so the patients don’t really need any written instruction.

**9. Lin 240, please revise “the effect between 239 suprathreshold and subthreshold
10. 240 stimulation will be investigated using two Verum groups.”**

We rephrased this sentence.

**11. What is CE in “CE-certified” Line 247**

CE means “Conformité Européenne” and is the european equivalent to "FDA approved".

**12. In PLOS ONE protocol guideline, the authors would need to state how data saturation will be determined. Also, data availability statement is missing, please check PLOS ONE DATA policy.**

We are not sure what you mean with “data saturation”. Since this is just a study protocol paper, no data will be collected – so there is no data to be shared at this point. Once the study is finished and analyzed, the data will of course be mad available within the publication of the results (which will be another publication).

**13. I recommend registering this study on a research such as OSF.**

This is interesting, we never heard of this before. Thank you.
