# Supplementary material for: COVIVA: Effect of transcutaneous auricular vagal nerve stimulation on fatigue-syndrome in patients with Long Covid – A placebo-controlled pilot study protocol
Source: PLoS One. 2025 May 9;20(5):e0315606. doi: 10.1371/journal.pone.0315606 (PMC12063903; doi:10.1371/journal.pone.0315606)
Supplement: Attachment — Submitted filename: PONE-D-23-38498 Review.docx [file pone.0315606.s003.docx]

Line numbers in track-changes revised manuscript

Line 52: What does “implantation of a non-functional electrode” refer to? Implantation implies invasive device (yours is not).

Line 106: Would consider addressing the disparity between your statement “Existing research indicates that fatigue is partly triggered by increased levels of pro-inflammatory hormones known as cytokines in the blood, while its severity seems to correlate with the levels of these cytokines in the initial phase of the disease” with the fact that your enrolled patients are not in the acute phase of the disease. This implies prevention of chronic-fatigue would be better tackled by initiating therapy early in the acute setting of the disease to take advantage of the anti-inflammatory effect of taVNS.

Line 286: Do you mean that adverse events have not been previously reported? Would consider reviewing and addressing concerns raised by a recent meta-analysis looking at the safety profile of taVNS that stated “Repeated sessions and sessions lasting 60 min or more are also shown to be more likely to lead to AEs” (PMID 36543841). In particular, this should be addressed for patients that could over deliver even longer stimulation times than described in the protocol.

Line 306: type “able”

Line 360: Would describe this an optional cross-over design

Line 370: It is a little misleading to state “Several studies have shown that healthy patients undergo acute taVNS to investigate scientific questions in behavioral research [55–57]. None of the existing studies on this topic have reported any relevant side effects.” While generally well-tolerated, there are reported adverse events for taVNS, particularly for longer stimulation durations as you mention several lines later. Your enrolled patients are also not necessarily “healthy”, as you exclude some severe physical and mental comorbidities, but patients may have other confounding diagnoses that could impact the safety profile of your study.

As previous research has indicated VNS increases arousal, and this may certainly combat symptoms of fatigue, have you considered any directions to participants for timing of the intervention. IE performed during early hours of the day rather than just before bed?
