# Supplementary material for: COVIVA: Effect of transcutaneous auricular vagal nerve stimulation on fatigue-syndrome in patients with Long Covid – A placebo-controlled pilot study protocol
Source: PLoS One. 2025 May 9;20(5):e0315606. doi: 10.1371/journal.pone.0315606 (PMC12063903; doi:10.1371/journal.pone.0315606)
Supplement: Attachment — Submitted filename: Comments to the reviewers.docx [file pone.0315606.s006.docx]

Comments to the reviewers

We thank the reviewers for their valuable comments.

**Reviewer #2:**

**The revision is satisfactory, and I recommend the manuscript for publication. One of the reviewer evaluation criteria is "Have the authors described where all data underlying the findings will be made available when the study is complete?" The authors can consider adding such a statement. I noticed that there are two sections titled ‘Study Plan’ in the manuscript. These changes do not require another round of review.**

We changed the second “Study plan” to “Study procedure”. We also added a statement that the results will be published in peer reviewed journals.

**Reviewer #4:**

**1. In line: 214: It should be PCS score. The word “score” is missing.**

We corrected this accordingly.

**2. In line, 166 and 167 say that primary endpoint is measure by the average stimulation duration (which is a continuous variable). On the other hand, line 297 says that primary outcome is an incidence rate with patients who exceed a usage-time-threshold (which is a binary categorical variable). The definitions need to be consistent. See also the definition of primary end point in the discussion section of the abstract.**

This is correct, we changed that.

**3. In line 218, which test statistics was used to calculate the sample size of 36 needs to be mentioned.**

We added the respective information.

**4. In line 223, It is better to mention that “other” secondary measures BDI, SF36 and PCSS……. This is because the first secondary measure MFI20 is already mentioned.**

Corrected.

**5. Sample size calculation section only has got the reference for sample size calculation ideas but not the exact sample size calculation for the trial. On the other hand, Data analysis section has got sample size calculation, statistical analysis as well as definition of end points. It would be better if clear definitions of primary and secondary end points are moved to the end points section. Similarly, sample size calculation and statistical analysis sections separated for clarity of presentation.**

We corrected that, too.

**Reviewer #5:**

**1. The manuscript describes the use of Fisher’s exact test and Kruskal-Wallis tests, but the rationale for selecting these statistical methods over alternatives should be explicitly justified, particularly in comparing the three groups.**

This was added.

**2. The sample size calculation is based on prior taVNS studies, but there is no clear power analysis for the primary endpoint. Clarify whether a sensitivity analysis was conducted to account for expected dropout rates and compliance variability.**

The power analysis is now in line 208. The drop-out-rate was estimated with the numbers of Redgrave et. Al. 2018 in mind, stating that throughout all studies in the meta-analysis around 3% of all participants drop out. In patients with long-covid we estimated this number a bit higher.

**3. The sham control uses a non-functional electrode, but prior research suggests that even subthreshold stimulation may induce physiological effects. A stronger justification for this approach or a discussion on potential placebo effects and blinding validation would strengthen the study design.**

We have three groups: Sham, Verum-1 and Verum-2. Verum-2 is standard stimulation. Verum-1 is stimulation below the threshold, but still a stimulation. Sham is no stimulation at all, guaranteed by a non-functional plastic-electrode with no stimulation capability. This is mentioned in line 298.

**4. The protocol allows up to 4 hours of daily taVNS, yet studies indicate that prolonged stimulation (>60 minutes) may lead to side effects such as headaches or nausea. The authors must comment on this.**

We added a respective sentence. However, the stimulator we use has a CE-certificate as a medical product in Germany and is certified for recommended use of 4h/day. Both software and integrated timer are calibrated to this recommendation.

**5. Since taVNS influences arousal and autonomic function, the timing of stimulation (e.g., morning vs. evening) could impact outcomes. Clarify whether stimulation timing should be standardized or at least documented to analyze its effects on fatigue and QoL.**

Thank you, this is an interesting point, indeed. To our knowledge, no study has so far addressed this aspect, also the manual of the stimulator does not recommend any specific time. However, the time of stimulation is logged both by the app and the patient’s stimulation diary and will consider analyzing this aspect.
